# Myogenesis and Analysis of Antimicrobial Potential of Silver Nanoparticles (AgNPs) against Pathogenic Bacteria

**DOI:** 10.3390/molecules28020637

**Published:** 2023-01-07

**Authors:** Palwasha Hayat, Ibrar Khan, Aneela Rehman, Tayyaba Jamil, Azam Hayat, Mujaddad Ur Rehman, Najeeb Ullah, Abid Sarwar, Amnah A. Alharbi, Anas S. Dablool, Zubaida Daudzai, Abdulhakeem S. Alamri, Majid Alhomrani, Tariq Aziz

**Affiliations:** 1 Department of Microbiology, Abbottabad University of Science & Technology, Havelian 22010, Pakistan; 2 Food and Biotechnology Research Center, PCSIR Labs Complex Lahore, Lahore 54600, Pakistan; 3 Department of Biochemistry, Faculty of Science, University of Tabuk, Tabuk 71491, Saudi Arabia; 4 Department of Public Health, Health Sciences College Al-Leith, Umm Al-Qura University, Makkah 24382, Saudi Arabia; 5 Department of Bioresource and Technology, King Mongkut’s University of Technology Thonburi, Bangkok 10140, Thailand; 6 Department of Clinical Laboratory Sciences, The Faculty of Applied Medical Sciences, Taif University, P.O. Box 11099, Taif 21944, Saudi Arabia; 7 School of Food & Biological Engineering, Jiangsu University, Zhenjiang 212013, China

**Keywords:** myogenesis, silver nanoparticles, *Penicillium notatum*, MDR, XDR, EDX, SEM1

## Abstract

The widespread and indiscriminate use of broad-spectrum antibiotics leads to microbial resistance, which causes major problems in the treatment of infectious diseases. However, advances in nanotechnology have opened up new domains for the synthesis and use of nanoparticles against multidrug-resistant pathogens. The traditional approaches for nanoparticle synthesis are not only expensive, laborious, and hazardous but also have various limitations. Therefore, new biological approaches are being designed to synthesize economical and environmentally friendly nanoparticles with enhanced antimicrobial activity. The current study focuses on the isolation, identification, and screening of metallotolerant fungal strains for the production of silver nanoparticles, using antimicrobial activity analysis and the characterization of biologically synthesized silver nanoparticles by X-ray diffraction (XRD) spectroscopy, energy-dispersive X-ray spectroscopy (EDX), and scanning electron microscopy (SEM). In total, 11 fungal isolates were isolated and screened for the synthesis of AgNPs, while the *Penicillium notatum* (K1) strain was found to be the most potent, demonstrating biosynthetic ability. The biologically synthesized silver nanoparticles showed excellent antibacterial activity against the bacteria *Escherichia coli (ATCC10536)*, *Bacillus subtilis*, *Staphylococcus aureus (ATCC9144)*, *Pseudomonas aeruginosa (ATCC10145)*, *Enterococcus faecalis*, and *Listeria innocua (ATCC13932)*. Furthermore, three major diffraction peaks in the XRD characterization, located at the 2θ values of 28.4, 34.8, 38.2, 44, 64, and 77°, confirmed the presence of AgNPs, while elemental composition analysis via EDX and spherical surface topology with a scanning electron microscope indicated that its pure crystalline nature was entirely composed of silver. Thus, the current study indicates the enhanced antibacterial capability of mycologically synthesized AgNPs, which could be used to counter multidrug-resistant pathogens.

## 1. Introduction

Antibiotics are generally recognized as antimicrobial agents that have the potential to control microbial infections. In the medical field, antibiotics have been in use for decades [1]. There are many different types of antibiotics available on the market that are used as medications for very complicated and severe diseases. Antibiotics are extracted from a variety of microbiological sources and are designed to target specific functions of the cell to inhibit microbial growth [2]. However, self-medication, over-dosage, low socio-economic status, and improper health education are the major reasons for an increasing trend of antibiotic resistance. Nowadays, antibiotic resistance has become a worldwide risk that has not only resulted in an increased death rate and deadly infection but also the emergence of multidrug-resistant pathogens. Currently available antibiotics are temporary solutions to various deadly infections; therefore, novel approaches are needed to overcome the menace of microbial resistance. 

Nanotechnology is an evolving multidisciplinary science that involves biology, physics, chemistry, material science, and medicine [3]. As a result of advances in nanotechnology, new vistas in nanomedicine have opened up, allowing for the manufacturing of nano-antibiotics/nanoparticles. Nowadays, nanoparticles (NPs) are used in a wide range of sectors, such as manufacturing and materials research, environmental science, energy, cancer treatment, gene therapy, drug delivery, tissue engineering, in vivo imaging, and the medical sector [4,5]. These nano-antibiotics have several advantages compared to conventional antibiotics, such as absorption, controlled release, targeted delivery, durability, and quick, targeted circulation, and are economical and highly effective against resistant microbial pathogens. These nanoparticles have garnered interest in the medical and health sectors because they not only possess antimicrobial activities but can also be used to enhance the efficacy of commonly used antibiotics [5]. 

Nanoparticles (NPs) are ultrafine units with dimensions that are measured in nanometers, ranging from 1–10^−9^ nm [2]. These small-sized particles show dramatic changes and novel properties that result in their application in various fields. The size, shape, surface topology, and different functional groups are important aspects that determine the specific properties of nanoparticles. There are numerous physical, chemical, biological, and hybrid methods for the synthesis of various types of nanoparticles (NPs). The biggest issue with the chemical and physical methods of silver nanoparticle manufacturing is that they are extremely expensive and also necessitate the use of toxic substances. These conventional methods of manufacture also result in potential environmental and biological dangers [6]. As these conventional approaches are expensive and have numerous limitations, scientists are thus working on developing clean, economical, and environmentally friendly biological approaches as an alternative method for NP synthesis [7].

Nanoparticles are biologically synthesized using various microorganisms that result in environmentally friendly, long-lasting, bio-functional, and durable nanoparticles. Among the microbes, bacteria, fungi, yeast, and actinomycetes have been reported to produce bio-functional nanoparticles that have wide-ranging applications in the health and medical sectors. The types of microbes and bioprocesses have a significant effect on the synthesis of nanoparticles. Among the microbes, bacteria and fungi are a preferred source for synthesizing nanoparticles, due to their physiology, established metabolism, and partial understanding of synthesis mechanisms. Mycogenesis is a most beneficial and new domain for nano-antibiotics synthesis because it results in the economical and bulk production of these biologically active substances within a very short period of time [8]. Moreover, fungi can be easily handled and cultured and are less prone to metal toxicity. Nanoparticles are synthesized by fungi in both intra- and extracellular environments. For advanced and modern scientific and medical research, nanoparticle synthesis utilizing fungi is becoming increasingly important due to the various qualities of nanoparticles, such as the size, structure morphology, targeted delivery, and stability of nanoparticles [9]. 

Biosynthesized nanoparticles have also been reported as potential pesticides for the treatment of a variety of planted plant and fungal ringworm in laboratory studies. In order to attain sustainable food production, nanoparticles have the potential to be widely used in food production as a biocontrol. Among nanoparticles, AgNPs have been reported for a wide range of antimicrobial activity against various plant and human pathogens. Moreover, AgNPs in the form of inclusion are used in wound healing and dressings. Biologically synthesized AgNPs have multiple applications in various sectors, such as medicine, infection control, food, pharmaceutical, cosmetics, etc. The fact that bacterial resistance to elemental silver is extremely rare emphasizes the increased interest in using AgNP as a potent antimicrobial agent in biomedical applications. Nowadays, fungi are the preferred source for biogenic nanoparticle synthesis because fungi are more metal-tolerant, can be easily handled, and can produce bulk quantities of the desired product.

Thus, this study aims to achieve the synthesizing and characterization of AgNPs of natural origin by utilizing heavy metal-tolerant fungal strains that could be used in a variety of applications, including biocontrol in the form of pesticides, potent antimicrobial substances, pollution control, etc. 

## 2. Results and Discussion

Nanotechnology is a multidisciplinary field that involves the production of valuable nanoproducts on a physical, chemical, and biological scale, as well as their utilization on a larger scale in various sectors. Various types of nanoparticles are commonly synthesized and utilized for numerous biological functions, such as copper (Cu) [10], gold (Au), iron (Fe), and silver (Ag). Silver nanoparticles (AgNPs) are ultrafine units with dimensions measured in the range of 1–100 nm [11,12]. Traditionally these nonantibiotics were synthesized using different physical and chemical methods. Non-ecofriendly, toxic, expensive, and limited yields are a few of the drawbacks associated with traditional methods; therefore, new approaches to NP synthesis must be designed to overcome the consequences of these previously used methods [13]. Nowadays, nanoparticle synthesis is practiced using biological sources. Microorganisms are the most effective source for the synthesis of silver nanoparticles because their physiology and metabolism are already known. Moreover, microbial synthesis results in bulk production and less hazardous manufacturing processes [14,15].

### 2.1. Isolation of Metal-Resistant Fungal Strains

In the current study, a total of 11 fungal strains were isolated from heavy metal-contaminated soil and then cultured on potato-dextrose agar (PDA) to determine their potential for growth. All 11 strains were used in the synthesis of silver nanoparticles; the K1 strains showed the highest ability to synthesize silver nanoparticles, compared to other strains. Thus, the k1 strains were selected for further study.

### 2.2. Identification of k1 Strain

#### 2.2.1. Microscopic Identification

**Figure 1:**
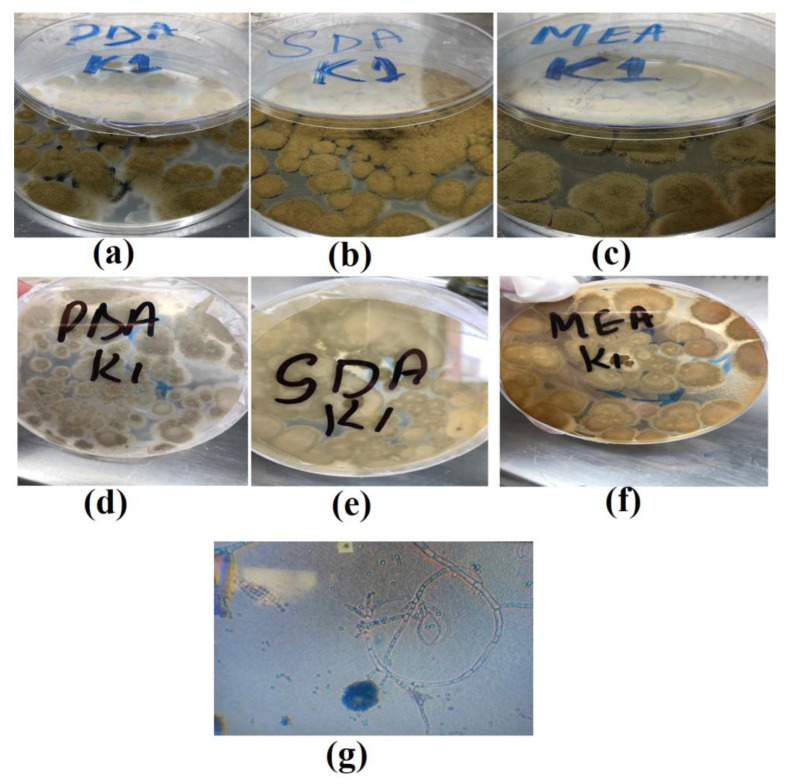
Morphological identification of the K1 strain on PDA, SDA, and MES media ((**a**–**c**) front; (**d**–**f**) back; (**g**) the chains of mycelium and spores).

The morphological identification of the selected fungal isolate was performed using potato-dextrose agar (PDA), Sabouraud dextrose agar (SDA), and malt extract agar (MEA) media at 30 °C for 7 days. The K1 strain colonies cultured on PDA medium showed rapid development, with the upper portion of the colonies being olive green in color (Figure 1a) and were radially sulcate to plicate, whereas the lower portion of the colonies was a dull white in color (Figure 1d), while the colonies on the SDA plates showed light green characterization with a velutinous (soft and velvety) surface texture (Figure 1b). Completely brown colonies were found with soluble pigments, while the reverse side of the colonies was found to be yellowish in color (Figure 1e). The K1 strain that was cultured on MEA plates displayed moderate growth, velvety smoothness, parrot-green frontal sides, sporangial hyaline hyphae, branching conidiophores, and phialides arranged in branching filamentous clusters (Figure 1c). The reverse side of the colony was white (Figure 1f). Upon microscopic analysis, the hyphae were found to be sporangial and hyaline and the conidiophores were observed to have a branched structure, indicating that the K1 strain has many characteristics similar to *Penicillium* (Figure 1g). A similar study by Gautam et al. [16] could be correlated with the present study; the authors isolated the metallotolerant fungal species *R. pusillus*, *A. flavus*, *A. terreus*, *A. tubingensis*, and *N. hiratsukae* from a Pb- and Cr-contaminated industrial site.

#### 2.2.2. Molecular Identification

**Figure 2:**
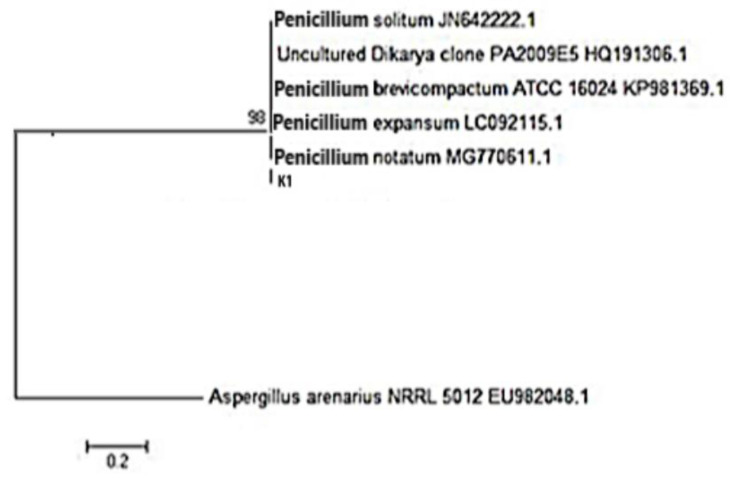
The phylogenetic tree of the fungal isolate, constructed using a neighbor-joining analysis of ITS.

A PCR product of 541 bp was obtained by isolating the DNA of the fungal isolates during the entire genomic process, utilizing DNA as a template. The ITS sequences of the fungal isolate were aligned, and a phylogenetic tree was constructed. The similarity of ITS between the isolated fungal strain and NCBI record showed that the isolate strains used in this study showed a similarity to *Penicillium notatum* (Figure 2). A similar study was conducted by Khan et al. [17], in which the authors identified different fungal species on the basis of colony morphology on differential media (SDA, PDA, and MEA) and ITS analysis, which showed these isolates as: M1 (*Aspergillus niger* DGR1), M3 *(Aspergillus fumigatus* Ai2), M6 (*Aspergillus terreus* 1), and M7 (*Aspergillus flavus*).

### 2.3. Synthesis of Silver Nanoparticles

A total of 11 metallotolerant fungal isolates were grown in a broth medium (potato dextrose broth). Each flask was incubated in an orbital shaker at 28 °C and 150 rpm. After seven days, the supernatant was removed via filtration, and a salt solution of silver nitrate AgNO_3_ was added to the filtrate. The flask was incubated at 32 °C in a shaker at 200 rpm. Additionally, under the same conditions, a positive control (filtrate and salt solution) and a negative control (culture filtrate) were maintained. The appearance of brown coloration in the reaction vessels showed the synthesis of nanoparticles. Among all the tested fungal strains, K1 showed the maximum production of AgNPs (1.9 mg) (Table 1). The change in color during the experiment can be interpreted as an indicator of silver nanoparticle synthesis. A surface plasmon vibration excitation in the silver nanoparticles was hypothesized to be the cause of the production of brown coloration in the silver nanoparticles. Moreover, it has also been documented that NADH-dependent reductases are involved in the synthesis of metal nanoparticles. The current findings are in accordance with the study conducted by Rajput et al. (2016), who reported silver nanoparticle synthesis in a liquid medium via a color change that is associated with the reduction of silver ions to silver nanoparticles.

Similar findings for the production of AgNPs using metallotolerant fungal strains were also reported in a study conducted by Velusamy and coworkers [18].

### 2.4. Antimicrobial Activity of Silver Nanoparticles

The well diffusion method was used to determine the antibacterial activity. Different concentrations of silver nanoparticles (1000 g, 500 g, 250 g, and 125 g) were used to check the antibacterial activity of AgNPs. The zone of inhibition against each bacterial species was checked after 24 h of incubation. The activity of *E. coli* in dilutions with various concentrations (1000 µg, 500 µg, and 250 µg) of silver nanoparticles was observed as 3 mm, 2.5 mm, and 0 mm, respectively. The antibacterial activity of AgNPs against *E. coli* was also reported by earlier scientists, when the zone of inhibition was observed as 4 mm, 4.5 mm, and 3 mm, respectively [19]. The activity of *Bacillus subtilis* in dilutions with various concentrations (1000 µg, 500 µg, and 250 µg) of silver nanoparticles was observed as 1.3 mm, 0 mm, and 0 mm, respectively. The inhibitory effects of AgNPs against *B. subtilis* were also reported in earlier studies conducted by Inagaki et al. [20]. Similarly, when the activity of *Cronabacter sakazaki* in dilutions at various concentrations (1000 µg, 500 µg, and 250 µg) of silver nanoparticles was checked, no zone of inhibition was observed. This indicated the resistance of the mentioned bacterial strains against biologically synthesized AgNPs. The current findings are in contrast with the results published by Chapman et al., who reported the highly effective activity of AgNPa against *C. sakazaki*. The antibacterial activity of *P. aeruginosa* with various concentrations (1000 µg, 500 µg, and 250 µg) of silver nanoparticles was observed as 1.2 mm, 0 mm, and 0 mm, respectively. The results of the study by Shang, et al. showed that AgNPs had a highly bactericidal effect on drug-resistant or multidrug-resistant *P. aeruginosa* [21]. When the antibacterial activity of AgNPs was checked against *Enterococcus faecalis*, zones of inhibition of 3.2 mm (1000 µg) and 1.7 mm (500 µg) were observed, whereas *Listeria innocua* showed sensitivity against all the tested concentrations 2.5 mm (1000 µg), 1.5 mm (500 µg), and 1.0 mm (250 µg) (Table 2). The antibacterial activity of biologically synthesized nanoparticles against *Enterococcus faecalis* and *Listeria innocua* was reported in studies conducted by the authors of Shahverdi et al. [12] and Inagaki et al. [20] The antibacterial activity of AgNPs against a large group of tested bacterial strains shows that they possess a small size and are, therefore, readily absorbed by the cells and show toxic effects. The common mechanisms reported by the antimicrobial activity of NPs include the inactivation of the cell wall’s sulfhydryl groups, the formation of insoluble compounds, and the disruption of cell-wall lipids and enzymes, which ultimately results in cell disruption [22]. It is also reported that nanoparticles synthesize special pores, via binding with surface proteins, which facilitate the direct targeting of the DNA replication mechanism.

### 2.5. Characterization of Microbially Synthesized AgNPs

The biologically synthesized NPs were subjected to different characterization techniques, in order to evaluate their potential.

#### 2.5.1. Structural Characterization of AgNPs with X-ray Diffraction (XRD)

**Figure 3:**
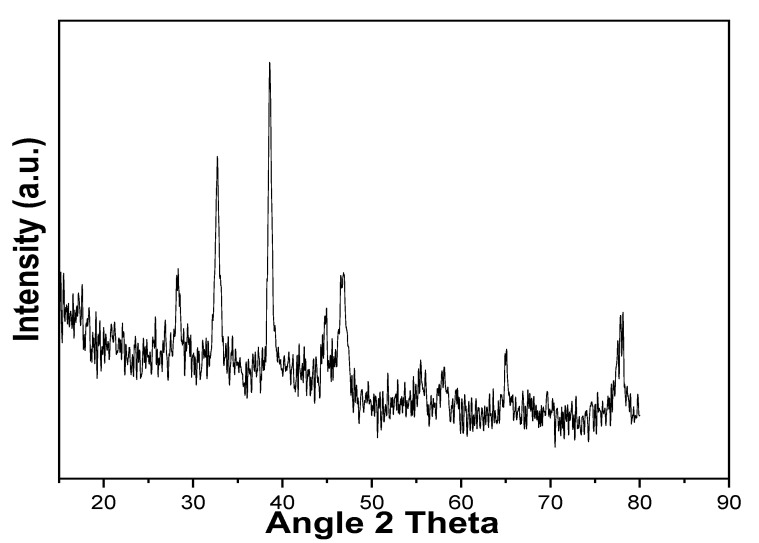
XRD analysis of the silver nano-powder.

XRD analysis reveals the crystallinity and purity of the synthesized nanoparticles. In the current study, the XRD spectrum matches well with the Ag results. No extra peak was observed, which confirms the presence of pure single-phased Ag. The presence of AgNPs is confirmed by the occurrence of three large diffraction peaks at the 2θ values of 28.4°, 34.8°, 38.2°, 44°, 64°, and 77°, which further confirm the synthesis of pure silver nanoparticles via mycogenesis (Figure 3). Our results are in accordance with the findings of Tyagi et al., who reported the biological synthesis of AgNPs using entomopathogenic fungi (*Beauveria bassiana*). The characterization of AgNPs via EDS and XRD also confirmed the crystalline nature of synthesized nanoparticles [23].

#### 2.5.2. Elemental Diffraction X-ray Spectroscopy (EDX)

**Figure 4:**
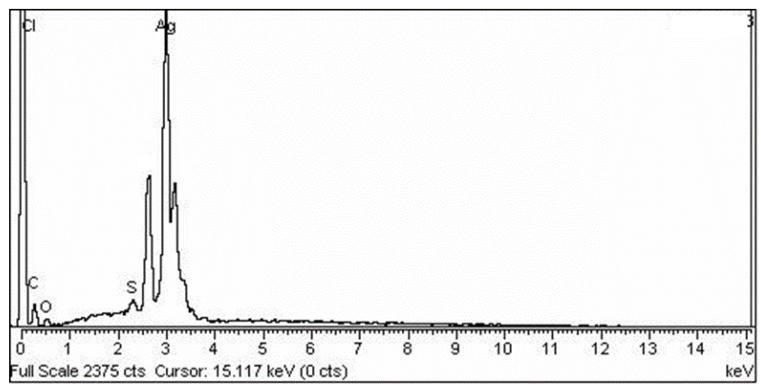
EDX analysis of the silver nano-powder.

EDX is used to determine the elemental position of a sample. The elemental composition analysis conducted by EDX showed the strongest signal for the silver (Ag) region, along with weaker signals from the Cl, O, P, Na, and Ca atoms. It also showed that its pure crystalline nature was solely composed of silver (Figure 4). The biogenic synthesis of AgNPs was carried out using the *Bacillus* strain CS11. The characterization of bacterial nanoparticles via EDX indicated that these nanometal particles showed the strongest signal for the silver region [23].

#### 2.5.3. Scanning Electron Microscopy

**Figure 5:**
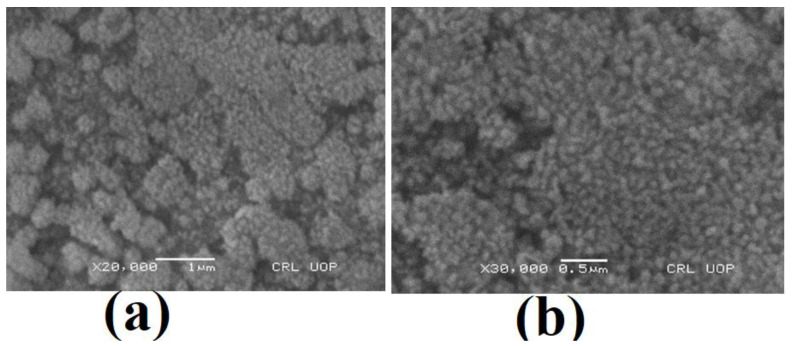
SEM micrographs of the AgNP nanopowders at (**a**) 1 micrometer and (**b**) 0.5 micrometer.

The surface topology of the synthesized AgNPs was studied using scanning electron microscopy. The micrographs shown in Figure 5 demonstrate that the synthesized NPs have a spherical shape and also show agglomeration. Agglomeration is a common phenomenon observed in AgNPs to achieve stability. The average size of spherically shaped particles is around 150 nm, but particles with a size of 200 nm were also found. Along with instability and agglomeration, a wide range of particle sizes is another major issue; while dealing with NPs, the surface of the particles appeared rough, with irregular boundaries and an asymmetric texture Figure 5. The surface topology of AgNPs synthesized by *Penicillium notatum* was also reported earlier via SEM by Bantan and coworkers Abu-Hamdeh, Nusier, and Karimipour [18].

## 3. Materials and Methods 

### 3.1. Sample Collection

Soil samples were collected in sterile and labeled polythene homogenizing containers from an industrial effluent collecting point. The soil samples, contained in homogenized polyethylene bags, were sealed tightly and transported back to the laboratory for the pretreatment and analysis of the samples. Two weeks after being collected, the contaminated soil samples were air-dried under laboratory conditions (in an oven at 75 °C) and stored in desiccators for further examination [17].

### 3.2. Isolation of Metal-Resistant Fungal Strains

The isolation of indigenous fungal strains from soil samples was carried out via the serial dilution method, using Sabouraud dextrose agar (SDA). In this study, samples were diluted up to 10^−6^ times, utilizing the serial dilution method. A sample (1 mL) from each of the dilutions by 10^−4^ to 10^−6^ times was plated onto SDA plates that were supplemented with 0.55 gL^−1^ chloramphenicol. The plates were incubated at 28 °C for 96 h after inoculation. The colonies of the most prevalent fungal genera were picked up and purified using the streak-plate approach [24].

### 3.3. Identification of Resistant Fungi

#### 3.3.1. Morphology-Based Identification 

Different growth mediums, such as potato dextrose agar (PDA), Sabouraud dextrose agar (SDA), and malt extract agar (MEA), were used to visualize the morphological differences. Under a fluorescence microscope, young developing fungi (2 days old) of each strain from the different mediums were collected and observed, using the cellSens standard software. Pure fungal strains were isolated and purified on PDA media for further preservation purposes [25].

#### 3.3.2. Molecular Identification

The total genomic DNAs of the fungal isolates were isolated and purified using the method described by Khan, Aftab, Shakir, Ali, Qayyum, Rehman, Haleem, and Touseef [24]. PCR amplification of the ITS conserve sequence was conducted using primers consisting of IT5:5′-TCCGTAGGTGAACCTGCGG-3′ and IT5:5′-TCCTCCGCTTATTGATATGC-3′ [24] from different fungal species that remained untouched, in order to conduct a genetic analysis between several unique fungal strains observed at the National Center for Biotechnology Information (NCBI). This method required 25 uL of the reaction mixture, which contained 12.5 u taq of DNA polymerase, 2.5 uL. of buffer (10×), 0.8 mM of dinitro triphosphate (dNTP), 1.25 uL of the DNA template, and 16.6 uL H_2_O for the PCR process. Process conditions include denaturation at 94 °C for a total of 10 min, with an early denaturation at 94 °C for 1 min, annealing at 53 °C for 1 min, an extension at 72 °C for 2 min, and a final extension at 72 °C for 10 min. A total of 32 cycles of PCR were performed in this experiment. To separate the PCR products, 1% agarose gel was used during the electrophoresis. The TIANgel Midi Filtration kit (TIAGEN BIOTECH, Beijing, China) was used to create gel bands containing the undesired product, after which a sequencing procedure was carried out on the gel bands.

Using BLAST analysis, the above-mentioned sequences were concatenated to form phylogenetic relationships, which were then analyzed further. Sequences that were found to be 98% similar to the currently known sequences were considered to be from the same species. Furthermore, Clustal X 1.83 was used to perform numerous alignments, and MEGA 4.0 was used to construct the phylogenetic relationships between the taxa.

### 3.4. Myogenesis of Silver Nanoparticles (AgNPs)

#### 3.4.1. Preparation of Biomass 

AgNP myogenesis was carried out using metallotolerant fungal isolates. A broth medium that contained 3 g/100 mL of malt extract, 100 mL of glucose, 3 g of yeast extract, and 5 g/100 mL of peptone was used for the aerobic growth of fungal isolates. The fungal culture was incubated at 28 °C in an orbital shaker spinning at 150 rpm. After 96 h of incubation, filtration was carried out with Whatman filter paper number 1, then the fungal biomass was completely removed using centrifugation and filtration. The filtrate from the fungal culture was employed to synthesize the nanoparticles. 

#### 3.4.2. Biosynthesis of Silver Nanoparticles

First, 1 mM (0.017 g/100 mL) of silver nitrate solution was prepared, then 50 mL of the solution was mixed with 50 mL of filtrate. The reaction was carried out in complete darkness at 28 °C for 96 h (at 150 rpm). The biosynthesis of AgNPs was checked after 12 h, 24 h, 48 h, and 72 h. A positive control (culture filtrate without the precursor salt) and a negative control (only silver nitrate solution) were run in parallel with the experimental flasks, to ensure that the results were accurate [26].

### 3.5. Antimicrobial Activity of AgNPs

The antibacterial activity of the nanoparticles was examined against a variety of pathogenic bacterial clinical isolates, including *Escherichia coli* (ATCC10536), *Bacillus subtilis, Staphylococcus aureus* (ATCC9144), *Pseudomonas aeruginosa* (ATCC10145), *Enterococcus faecalis*, and *Listeria innocua* (ATCC13932). The antimicrobial activity of the biologically synthesized nanoparticles was analyzed using a well-diffusion essay. 

### 3.6. Characterization of AgNPs

The samples were characterized using scanning electron microscopy, elemental analysis, and X-ray diffraction analysis.

#### 3.6.1. XRD

The XRD measurements of the pure powders of silver nanoparticles were performed using an XRD diffractometer (PANalytical-XPERT PRO diffractometer system), Malvern Panalytical Ltd., Malvern United Kingdom. The generator was operated at a voltage of 40 kV and a current of 30 mA. The scanning temperature range was set at between 10 and 100 °C. The Debye–Scherrer equation was used to determine the size of the crystallites. The XRD pattern was obtained at 30–80 °C. The Origin pro8 program was used to construct a graph [27].

#### 3.6.2. EDX

The samples were subjected to EDX analysis with the same instrument that was used for the SEM analysis, in order to confirm the elemental composition of the sample [28].

#### 3.6.3. SEM

For the SEM studies, samples were cast onto glass slides, followed by fixation on copper supports. The samples were covered with a thin layer of gold by sputtering. The coated surface was examined using a scanning electron microscope (SEM) (JEOL JSM 6360LA, Akishima City, Tokyo, Japan) that operates at 20–30 kV, in order to identify the morphology of the produced nano-silver and to determine the mean particle sizes. The scanning electron microscope is equipped with an energy-dispersive spectroscopy (EDS) unit for qualitative and quantitative analyses and elemental mapping for the produced nanoclusters.

## 4. Conclusions

Nanomaterials have achieved significant awareness in recent years, despite their numerous applications in the medical field (splints, prostheses, and opiates), groundwater remediation, textiles, agricultural (poison), and, probably most crucially, medications. In the current investigational study, *Penicillium notatum* was used for the production of silver nanoparticles. It has been discovered that *Penicillium notatum* produces the greatest quantity of nanoparticles after forty-eight hours of natural biogenesis; this production is reduced when using the microwave-aided biogenesis methodology (20 min). The average size of the nanoparticles, which was revealed to be 55–65 nm by SEM, EDX, and the XRD peaks, demonstrated that the nanoparticles have an FCC structure in nature. Their activity against diverse bacterial strains, as well as the zone of inhibition tests, have all demonstrated that the stability of AgNPs has improved over the course of history.

## Figures and Tables

**Table 1:** Concentration of nanoparticles synthesized from different fungal strains.

Strain	KI	K2	K3	K4	K5	K6	K7	K8A	K8B	K9	K10
AgNPs (mg)	1.9	1.1	0.9	1.5	1.0	1.2	0.7	1.66	1.7	1.3	1.23

**Table 2:** Pathogens with in vitro antibacterial activity on nanoparticle dilutions.

S#	Test Organism	Nature of Bacteria	Dilution	Zone Diameter
1.	*Escherichia coli*	Gram-negative	1000 µg	3 mm
			500 µg	2.5 mm
			250 µg	0
2.	*Bacillus subtilis*	Gram-positive	1000 µg	1.3 mm
			500 µg	0
			250 µg	0
3.	*Cronobacter Sakazakii*	Gram-negative	1000 µg	0
			500 µg	0
			250 µg	0
4.	*Pseudomonas aeruginosa*	Gram-negative	1000 µg	1.2 mm
			500 µg	0
			250 µg	0
5.	*Enterococcus faecalis*	Gram-positive	1000 µg	3.2 mm
			500 µg	1.7 mm
			250 µg	0
6.	*Listeria innocua*	Gram-positive	1000 µg	2.5 mm
			500 µg	1.5 mm
			250 µg	1.0 mm

## Data Availability

Not applicable.
